# Generalization of the Ratiometric Method to Extend pH Range Measurements of the BCECF Probe

**DOI:** 10.3390/biom13030442

**Published:** 2023-02-26

**Authors:** Alaa Tafech, Céline Beaujean, Yves Usson, Angélique Stéphanou

**Affiliations:** Université Grenoble Alpes, CNRS, UMR 5525, VetAgro Sup, Grenoble INP, TIMC, 38000 Grenoble, France

**Keywords:** fluorescence microscopy, BCECF, pH drift, cell culture medium, methodology

## Abstract

There is a variety of fluorescent probes for pH measurements and which are mainly used for biological systems. In general, they can be classified into two groups. The first group includes fluorescent pH probes which exhibit a single fluorescence emission peak. For these probes, the fluorescence excitation profile is pH-dependent and the shape of the emission spectra remains almost constant. Hence, the ratiometric pH measurement–which makes pH determination independent of the probe concentration-is implemented when the excitation is performed at two excitation wavelengths and the fluorescence emission is measured at one wavelength. The second group exhibits a dual fluorescence emission peak. Here, each protonated or deprotonated form exhibits characteristics emission and/or absorption spectra. Shifts between spectra obtained for protonated and deprotonated species can be exploited in order to perform a ratiometric measurement. In this study we present a methodology that evaluates the precision of the ratiometric measurements based on multiple wavelengths excitation to determine the optimum wavelengths combination for pH determination in biological samples. This methodology using the BCECF probe is applied to measure the pH drift in cell culture medium. It exhibits a high precision and significantly extends the range of validity for pH measurements spanning from very acidic to basic.

## 1. Introduction

At the end of the 19th century, fluorescent probes such as rhodamine, acridine orange and fluorescein, the most widely used in biology, appeared [1,2,3]. There are two categories, intrinsic and extrinsic probes. Intrinsic probes are those already present in the biological sample. These include, for example, aromatic amino acids, purines, pyrimidines, certain enzymatic cofactors (NADH) and vitamins [4]. While extrinsic probes are added for the analysis of a non-fluorescent compound, and serve to label a specific constituent [5,6]. Fluorescent molecules that intercalate with various molecules can be used to study enzymatic activity, cell viability or cell pH [7,8]. In this regard, there exists a variety of fluorescent pH probes that can be classified into two groups. The first group includes fluorescent pH probes which exhibit a single fluorescence emission peak (fluorescein for example [9]). For these probes, the fluorescence excitation profile is pH-dependent and the shape of the emission spectra remains almost constant. Hence, the ratiometric pH measurement is implemented when the excitation is performed at two excitation wavelengths and the fluorescence emission is measured at one wavelength. In other words, ratiometric pH measurement allows the observation of variations in the emission intensity ratio, hence the name ratiometric, by measuring the fluorescence at two separate excitation wavelengths. The second group exhibits a dual fluorescence emission peak (Carboxy SNARF-1 for example [10]). Here, each protonated or deprotonated form exhibits characteristics emission and/or absorption spectra. Shifts between spectra obtained for protonated and deprotonated species can be exploited in order to perform a ratiometric measurement. Two situations occur, either the excitation is performed at two excitation wavelengths and the fluorescence emission is measured at one wavelength, or the excitation is performed at only one excitation wavelength and fluorescence emission is measured at two emission wavelengths. Under certain conditions, ratiometric measurement makes pH determination independent of the probe concentration or optical path length because pH is related to the ratio (in excitation or emission) at two characteristic wavelengths.

The seminaphtarhodafluor (SNARF) family has shown interesting analytical properties for their applications in biological conditions [11,12,13]. Carboxy SNARF-1 was widely used from this family to monitor cell intracellular pH. It can be excited by the 488 or 514 nm spectral lines of the argon-ion laser. This advantage had made carboxy SNARF-1 suitable for both confocal laser scanning microscopy and flow cytometry. However, the relatively high pK${}_{a}$ (∼7.5) of carboxy SNARF-1 has limited its biological applications. This motivated to modify the existing SNARF-1 probe by fluorinating its benzo[c]xanthene ring system [14]. Therefore, a new fluorescent probe was synthesized and characterized as new dual-emission pH probe with pK${}_{a}$ values of ∼6.4. The low pK${}_{a}$ value of 6.4 for SNARF-4F makes this probe exceptionally suitable for pH measurement in the range from about 6.0 to 7.5.

**Figure 1:**
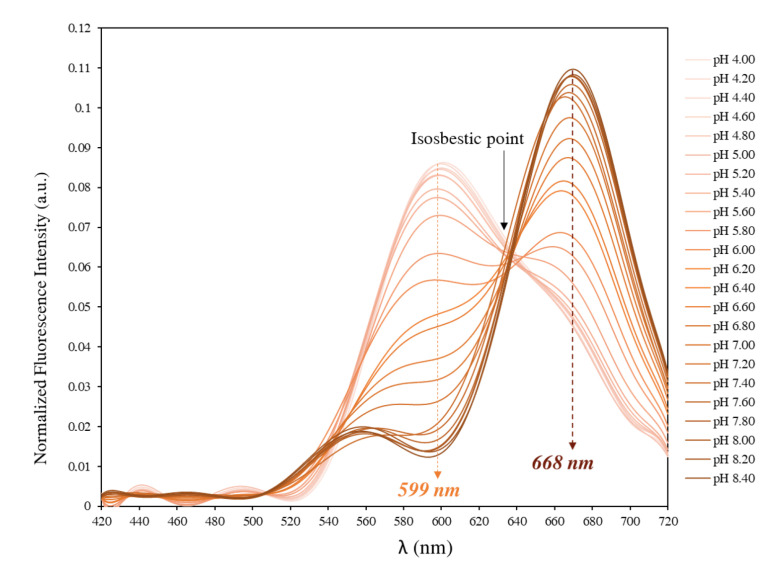
Emission spectra of SNARF-4F in DMEM serum-free medium at various pH values. SNARF-4F exhibits a double emission peak at 599 nm and 668 nm with an isosbestic point at 638 nm (pH-independent). Excitation wavelength is 514 nm.

When in solution, SNARF-4F exists under two forms: the acidic or protonated form and the basic or deprotonated form. Therefore, the fluorescence signal is due to the contribution of these two forms with relative contributions depending on the pH of the medium under test. Thus, at pH values well below its pK${}_{a}$, the probe exists predominately in a protonated state and exhibits maximum fluorescence emission at 580 nm when excited at 514 nm. Whereas, SNARF-4F exists in a predominately deprotonated state at pH values well above the pK${}_{a}$ and exhibits maximum fluorescence emission at 640 nm when excited at 514 nm in aqueous samples. We note that in Figure 1 the fluorescence emissions are 599 nm for the protonated form and 668 nm for the deprotonated form when excited at 514 nm in a cell culture medium, since the nature of the medium influences the emission wavelengths. Shift obtained between the spectra of the protonated and deprotonated species can be used to perform a ratiometric measurement for more accurate pH determinations. In this case, pH is directly related to the ratio of the fluorescence intensities measured at two emission wavelengths, typically about 599 nm and 668 nm. These two emission wavelengths can be minimally affected depending on the medium under test or even the excitation light used. Besides being a single excitation-dual emission fluorescent probe, SNARF-4F can be used as a dual excitation-single emission fluorescent probe. In such case, the excitation of SNARF-4F is performed at two excitation wavelengths and the fluorescence emission is measured at one emission wavelength, 599 nm or 668 nm. pH measurements are therefore made by determining the ratio of fluorescence intensities measured at both excitation wavelengths and fixed emission at 599 nm or 668 nm.

**Figure 2:**
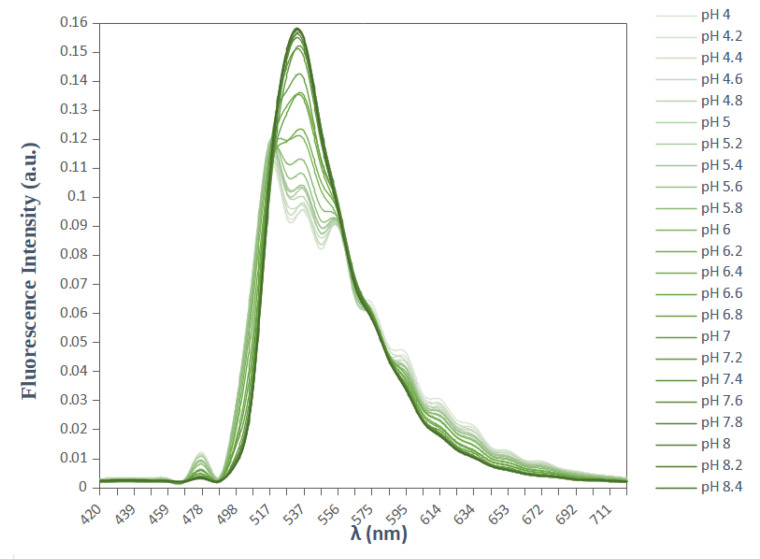
Emission spectra of BCECF in DMEM serum-free medium at various pH values. BCECF exhibits a single emission peak at 537 nm. Excitation wavelength is 488 nm.

The most widely used single fluorescence emission peak fluorophore for pH measurement is the BCECF. It was introduced by Tsien et al. in 1982 [15]. Based on a fluorescein core, it has the advantages of a high fluorescence quantum yield in basic medium (84%) and a pK${}_{a}$ equal to 7.0 adapted to physiological pH. The fluorescence intensity of the probe depends on the pH value, and it reaches a maximum amplitude for an emission wavelength at 537 nm when the probe is excited at about 488 nm (Figure 2). The shape of the emission spectra remains constant and the use of a single excitation wavelength does not make it possible to obtain an emission signal indicating only the variations in fluorescence linked to a change in pH. The decrease in fluorescence intensity can be the result of a bleaching of the probe following repeated excitations, or a direct consequence of the decrease in the concentration of BCECF due to leakage phenomena. In order to record only fluorescence variations due to pH variations, a second excitation wavelength is used, at 440 nm. The fluorescence intensity, at this wavelength, is independent of pH and reflects the probe concentration. Therefore, the use of the fluorescence ratio (R = F488/F440) makes it possible to avoid any variations in fluorescence independent of pH.

In the case of a confocal microscope, 440 nm Helium–Cadmium laser line is used for excitation in combination with the 488 nm Argon laser line. Thus, this combination allows pH measurement in the terms of the ratio of fluorescence intensities (R = F488/F440) measured at these two excitation wavelengths and fixed emission at 537 nm. Although the combination of these two excitation wavelengths is preconized, other configurations can be tested. We were brought to do it as our microscope was not equipped with the 440 nm Helium-Cadmium laser. As a dual-excitation probe, BCECF must be excited by two excitation wavelengths. Therefore, the first question that we asked was about the possibility of using another excitation wavelength, than the 440 nm laser, in combination with the 488 nm laser or even the possibility of using two other lasers, which do not even include the 488 nm, among the lasers we have. Hence the primary goal of this study was to find a method for pH measurement with BCECF that overcomes the material limitations of our confocal microscope.

To that end, the novelty of our approach has been to test all the available excitation wavelengths and to systematically calculate all the ratio combinations between the different emission wavelengths exhibiting pH-dependent fluorescent intensity. By this mean the best laser configuration has been identified. Although the preconized laser excitations for BCECF allow for pH measurements of about ±1 pH unit around pH 7, we were able with our generalized ratiometric method to significantly extend this pH range from 4 to 8.4 with an increased accuracy. This new method was then applied to compare the performance of SNARF-4F and BCECF to evaluate the experimentally reported pH drift in a cell culture medium.

## 2. Materials and Methods

### 2.1. Microscope

A Zeiss LSM 710 confocal microscope was used. It is equipped with the following 6 laser lines: Diode 405 nm, Argon 458 nm, 488 nm, 514 nm and Helium-Neon 543 nm, 633 nm.

### 2.2. Fluorescent Probes

Two probes with different properties were considered. SNARF-4F (SNARF-4F 5-(and-6)-carboxylic acid) which presents a double emission peak (with a single excitation) (Figure 1) and BCECF (2’,7’-bis(carboxymethyl)-5(6)-carboxyfluorescein) which presents a single emission peak (which requires a dual excitation) (Figure 2). Both probes were purchased from ThermoFischer Scientific. They were provided as lyophilized solids in units of 1 mg. Upon receipt, they were stored away from light at a temperature of −20 °C. Stock solutions were prepared at 0.1 mM in DMSO and then divided into 10 μL aliquots in microtubes (Eppendorf) stored at −20 °C.

### 2.3. Cell Culture Medium

The pH was measured in a DMEM free-serum medium, containing 4.5% glucose and 2 mM L-glutamine. The medium was maintained in a humidified atmosphere with 5% CO${}_{2}$ at 37 °C, which are the classical conditions used for standard cell cultures.

### 2.4. pH Adjustments for Calibration and pH Measurements

To calibrate the fluorescent probes, a large spectrum of pH for the culture medium were considered from 4 to 8.4 with 0.2 pH increments. The pH of the DMEM solution was measured with a pH meter and adjusted using 0.1M HCl or 0.1M NaOH solutions to the required value in atmospheric air, at room temperature. The solution was then immediately placed in the incubator at 5% CO${}_{2}$. pH measurement with the pH meter was realized by taking out of the incubator the DMEM solution for a few minutes. This time lapse is too short to significantly modify the pH from its value if it was kept in the incubator.

## 3. Results

### 3.1. In Vitro Fluorescence Spectra of SNARF-4F

**Figure 3:**
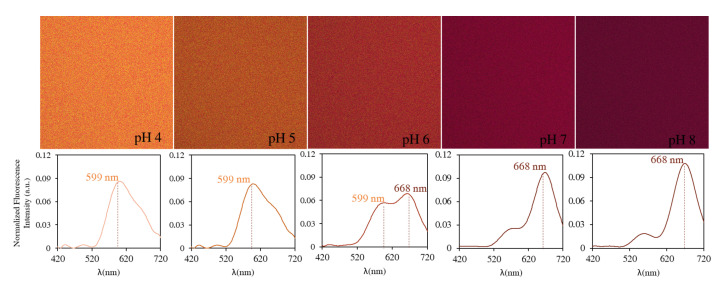
Fluorescence images of SNARF-4F in DMEM medium from pH 4.0 (left) to pH 8.00 (right) under 514 nm excitation. For each pH, we present the average emission spectrum over the entire image (2048 × 2048 pixels).

The fluorescence of the probe in the cell culture medium was examined using an excitation light at 514 nm provided by an Argon ion-laser and the fluorescence emission was recorded in the 420–720 nm range. As shown in Figure 1, when excited at 514 nm, SNARF-4F exhibits a pH-dependent wavelength shift with emission maxima at 599 nm and 668 nm, and an isosbestic point at 638 nm. In parallel, looking at Figure 3, the probe displayed a strong deep red fluorescence in neutral/basic medium ($\lambda_{em,max}$ = 668 nm). In contrast, when the pH of the medium was down-regulated to more acidic values, the fluorescence gradually changed from deep red to yellow-orange ($\lambda_{em,max}$ = 559 nm). Thus, increasing acidity was accompanied by yellow-shifts for emission spectra when the pH changed from neutral to acidic.

**Figure 4:**
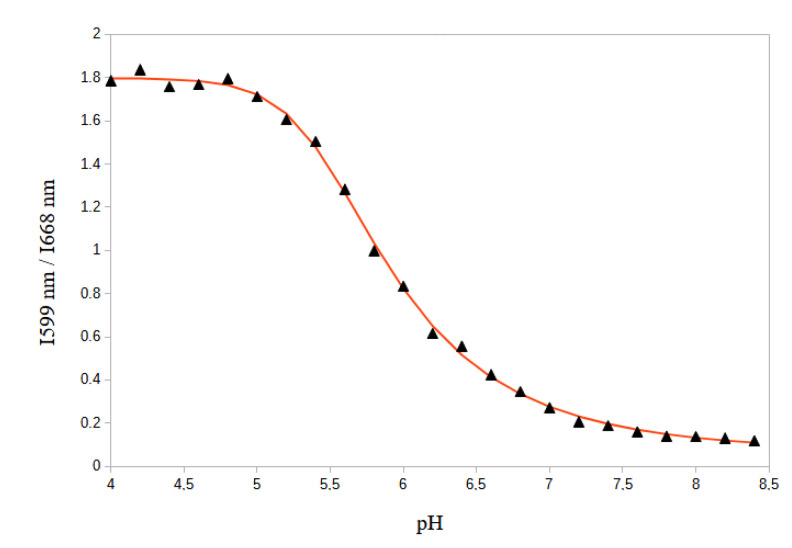
Ratio of fluorescence intensity (I${}_{599\mathrm{nm}}$/I${}_{668\mathrm{nm}}$) of SNARF-4F in DMEM serum-free medium at different pH values. The experimental points are fitted with a logistic regression function (continuous line) for which $R^{2}=0.9989$).

Therefore, as a dual emission probe, SNARF-4F can be used to deduce pH from the ratio of the fluorescence intensities emitted at the two wavelengths corresponding to the emission maxima. We found that the ratio between the fluorescence intensities at 599 nm and 668 nm (I${}_{599\mathrm{nm}}$/I${}_{668\mathrm{nm}}$) displayed a ∼15-fold enhancement (from 0.119 at pH 8.4 to 1.787 at pH 4.0) (Figure 4).

The experimental points are fitted with a five parameters logistic regression function that gives the relationship between the fluorescence ratio ($R=I_{559\mathrm{nm}}/I_{668\mathrm{nm}}$) and pH with a $R^{2}$ coefficient ($R^{2}=0.9989$). The regression function is given by:

(1) $$R=\frac{I_{559\mathrm{nm}}}{I_{668\mathrm{nm}}}=d+\frac{a-d}{{\left(1+{\left(\frac{pH}{c}\right)}^{b}\right)}^{m}}$$

with a=1.7952, b=23.0893, c=5.4754, d=0.0655, m=0.3710. Therefore,

(2) $$\mathrm{pH}=5.4754{\left[{\left(\frac{1.7298}{R-0.0655}\right)}^{2.6951}-1\right]}^{0.0433}$$

which allows to determine the pH with the best precision at the neighborhood of the SNARF-4F pKa (6.4) typically in the restricted range 6.2 to 7.2.

### 3.2. In Vitro Fluorescence Spectra of BCECF

**Figure 5:**
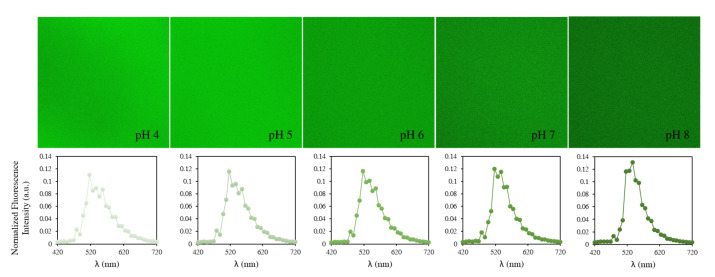
Fluorescence images of BCECF in DMEM medium from pH 4.0 (left) to pH 8.00 (right) under 405 nm excitation. For each pH, we present the average emission spectrum over the entire image (2048 × 2048 pixels).

**Figure 6:**
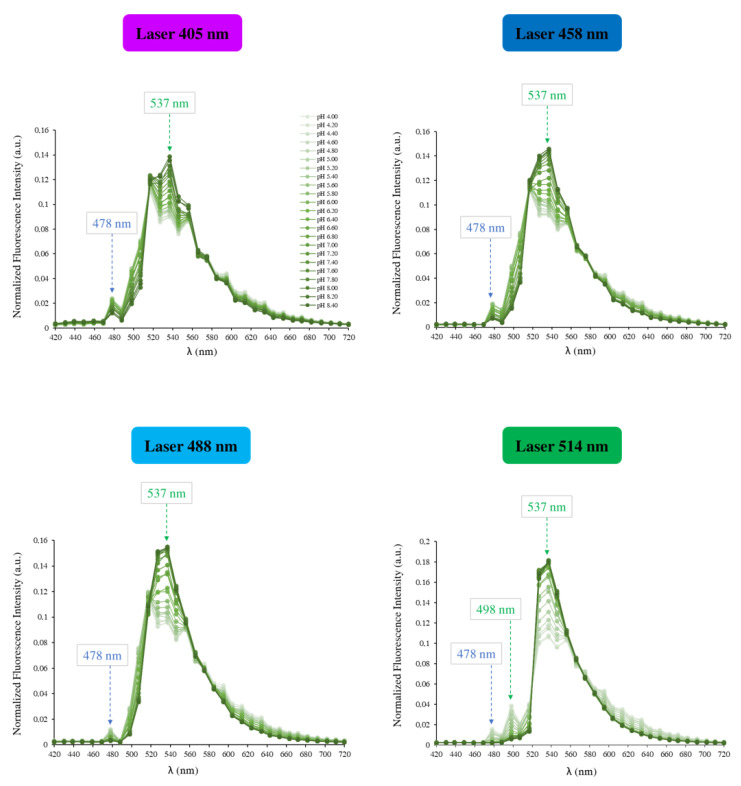
BCECF (5 μM) emission spectra at 405 nm, 458 nm, 488 nm and 514 nm excitation wavelengths, in DMEM free-serum medium at various pH values from 4.0 to 8.4 with 0.2 pH increments controlled with a pH meter. The pH-sensitive emission wavelengths assessed for the ratiometric measurements are represented by arrows.

As mentioned above, to ratiometrically measure the intracellular pH, BCECF is typically excited by two wavelengths: 440 nm and 488 nm. As we do not have the laser 440 nm, the goal was first to know if there is another excitation that can be used in combination with the 488 nm laser or whatever two other lasers among the lasers we have. Therefore, the first step of our study consists in finding among the lasers that we have those which can be used to excite the BCECF. To answer that, an initial experiment was performed in a serie of pH-adjusted DMEM free-serum medium, from 4.0 to 8.4 with 0.2 pH increments. The fluorescence of the probe was examined at each of the six lasers installed on our confocal microscope and the fluorescence emission was recorded in the 420–720 nm range. We found that among the six lasers, BCECF can be excited by four: 405 nm, 458 nm, 488 nm and 514 nm. Whether it is the excitation wavelength or the pH of the tested solution, the BCECF shows strong green fluorescence with a maximum emission intensity at around 537 nm (Figure 5). The emission spectra recorded at each excitation wavelength are shown in Figure 6.

The analysis of the emission spectra shows that the longer excitation wavelength gives the higher fluorescence intensities in all emission wavelengths we explored (420–720 nm). Namely, 514 nm produced the highest fluorescence values. Whatever the excitation wavelength, the fluorescence intensity of the probe is maximum when the emission wavelength is around 537 nm. Another emission wavelength with smaller fluorescence intensity, but common to all spectra, is noticed at 478 nm (blue arrow). The fluorescence intensities around 478 nm decrease when the pH increases, while those of around 537 nm increase at the same conditions. Only for the 514 nm laser, a third emission wavelength is noticed at 498 nm and responds to the pH changes similarly to 478 nm (green arrow).

**Figure 7:**
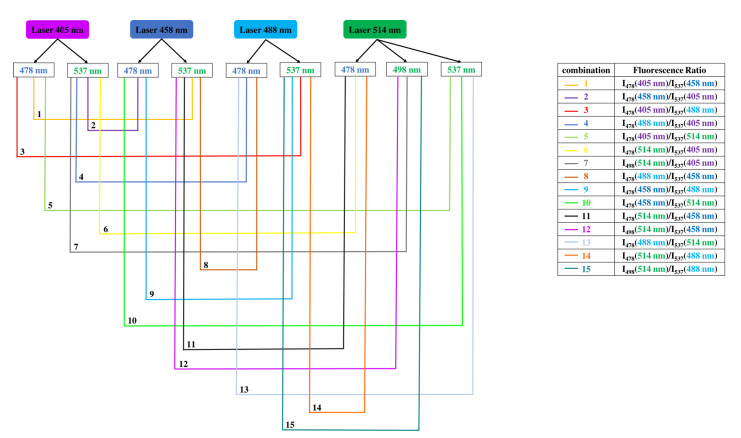
The 15 combinations made between one minor and one major emission wavelength of two different spectra of BCECF. For each combination, the ratio of fluorescence intensities measured at the two emission wavelengths is monitored as a function of pH.

As its name suggests, the ratiometric method is based on the use of a ratio between two fluorescence intensities measured at two emission wavelengths. A relationship between the pH and this ratio is therefore established. Starting from this principle, we measured the ratio between all the combinations of minor (478 nm and 498 nm) with major (537 nm) fluorescence emission intensities between the four different emission spectra of BCECF (Figure 6). The goal was to find a combination of two emission wavelengths allowing to detect the pH as a function of the fluorescence ratio. As the BCECF is used as a dual excitation probe, the two emission wavelengths are chosen separately from two different laser lines. In total, 15 combinations were made between the emission wavelengths. These combinations are shown in Figure 7. Every combination shows first the two laser lines used and therefore the emission wavelength chosen for each. For example the combination 1 (in yellow) shows that the two lasers used are 405 nm and 458 nm. For the 405 nm laser the emission wavelength is fixed at 478 nm while for the 458 nm laser the emission wavelength is fixed at 537 nm.

Once the two emission wavelengths are fixed, the ratio (*R*) of the fluorescence intensities measured at these two emission wavelengths is monitored as a function of the pH. This allows us to construct for each combination the curve *R* = *f*(pH). Above each curve is noted the corresponding combination. Statistically, two types of regression were used to estimate the relationship between the pH and the fluorescence ratio. The goal was to find a best-fit line that best expresses the relationship between the data points. To get an idea of how many data points fall within the results of the line formed by the regression equation, the coefficient of determination R${}^{2}$ or “R squared” was determined. The higher the coefficient, the higher the percentage of points crossed by the fit line. Among the 15 combinations, we found 4 combinations (1, 3, 5 and 10) with a coefficient of determination of 0.98 by applying a polynomial fit of degree 3. This means that 98% of the data points fall within the regression line. This higher coefficient is an indicator of a better goodness of fit for the observations.

**Figure 8:**
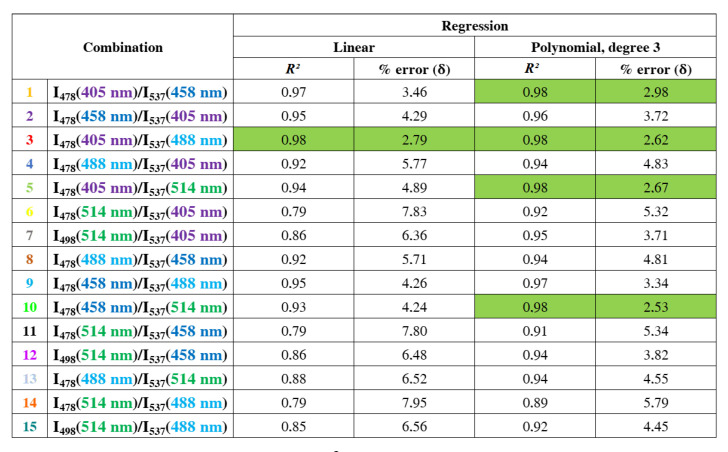
The coefficient of determination *R*${}^{2}$ and the percent error δ for each regression applied on the data points of each laser combination. In green, the 4 best combinations with a coefficient of determination *R*${}^{2}$ of 0.98 and a percent error smaller than 3%.

Since we take a measurement on the pH of the DMEM medium which has a reference value according to the pH meter, the percent error δ makes it possible to evaluate the importance of the difference between the pH value effectively measured and the reference value. It is recommended to have a percent error in the range of 0–3%. Figure 8 shows the coefficient of determination R${}^{2}$ and the percent error δ obtained for each regression made on the data points of each laser combination. The combinations 1, 3, 5 and 10 show, for a given regression (marked in green in Figure 8), a percent error lower than 3%. This confirms the choice for the best lasers combination with the highest coefficient of determination and the lowest percent error.

Four laser combinations meet the requirements that include a regression coefficient R${}^{2}$ closer to 1 and percent error smaller than 3%. We found that the 405 m laser can be used, instead of the 440 nm laser, in combination with the 488 nm laser for ratiometric measurements of pH. The originality of this study is based on the different possibilities of exciting the BCECF probe in the case of pH measurements. In the literature, only one method has been proposed to measure the pH with the BCECF probe. It consists of using the 440 nm laser in combination with the 488 nm laser and fixing the emission intensity at 537 nm. It is said that “some problems are opportunities and solutions to other problems”. Due to our material limitations, we have found several ways to excite the BCECF probe in order to ratiometrically measure the pH. The first way is to use the 405 nm laser in combination with the 458 nm laser and setting the emission wavelength at 478 nm for the 405 nm laser and at 537 nm for the 458 nm laser. In this case, a polynomial regression of degree 3 is applied on the data points ($R^{2}$ = 0.98, δ = 2.98%). The second way consists of using the 405 nm laser in combination with the 488 nm laser and setting the emission wavelength at 478 nm for the 405 nm laser and at 537 nm for the 488 nm laser. In this case, a well-fitting linear regression result was obtained for the observed data ($R^{2}$ = 0.98, δ = 2.79%). It is interesting to apply this excitation method since it is generally known that the fluorescence ratio is linearly related to the pH variation. The third way to measure the pH is to use of the 405 nm laser in combination with the 514 nm laser by setting the emission wavelength at 478 nm for the 405 nm laser and at 537 nm for the 514 nm laser. In that case, a polynomial regression of degree 2 is applied on the data points ($R^{2}$ = 0.98, δ = 2.66%). The last method allows us to use the 458 nm laser in combination with the 514 nm laser and setting the emission wavelength at 478 nm for the 458 nm laser and at 537 nm for the 513 nm laser. Here, a polynomial regression of degree 3 is applied on the data points ($R^{2}$ = 0.98, δ = 2.53%).

**Figure 9:**
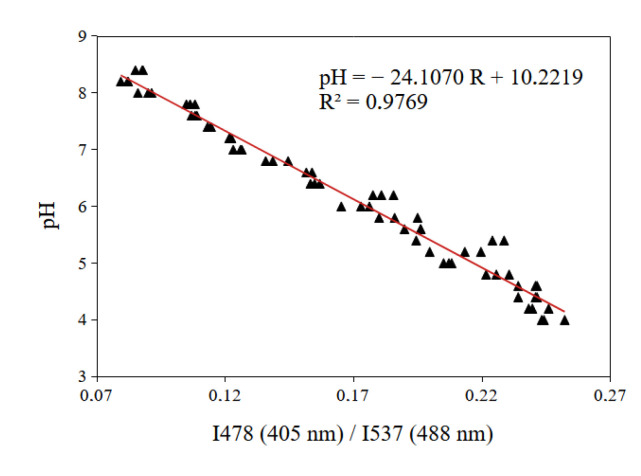
Calibration curve obtained from the best combination of the two lasers (405 nm and 488 nm) for BCECF. The emission wavelength was fixed at 478 nm for the 405 nm laser and at 537 nm for the 488 nm laser (data set in triplicate).

For our pH measurements, we chose the second method of excitation of the BCECF probe, which allowed us to apply a regression of smallest degree (linear) thus giving the lowest percent error. This method allows the implementation of the ratiometric pH measurement by exciting the pH probe with the 405 nm and 488 nm laser. The emission wavelength is fixed at 478 nm for the 405 nm laser and at 537 nm for the 488 nm laser. The calibration curve used to measure the extracellular pH is shown in Figure 9.

### 3.3. Application to the Measure of the pH-Drift in Cell Culture Medium

The bicarbonate buffer system is an acid-base homeostatic mechanism involving the balance of bicarbonate ion (HCO${}_{3}^{-}$) and carbon dioxide (CO${}_{2}$) to maintain a constant pH in the blood and duodenum, among other tissues, in order to support proper metabolic functions. Carbon dioxide (CO${}_{2}$) reacts with water (H${}_{2}$O) to form carbonic acid (H${}_{2}$CO${}_{3}$), which in turn rapidly dissociates to form bicarbonate ion (HCO${}_{3}^{-}$) and a hydrogen ion (H${}^{+}$) as shown in the following reaction [16,17,18]:

(3) $${CO}_{2}+H_{2}O⇌H_{2}{CO}_{3}⇌H^{+}+{HCO}_{3}^{-}$$

As with any buffer system, the pH is balanced by the presence of both a weak acid (e.g., H${}_{2}$CO${}_{3}$) and its conjugate base (e.g., HCO${}_{3}^{-}$) so that any excess acid or base introduced into the system is neutralized. CO${}_{2}$ stabilizes the pH at values compatible with cell activity, i.e., between 7.35 and 7.45 [19]. Failure of the buffer system leads to acid-base imbalance, such as acidemia (pH < 7.35) and alkalemia (pH > 7.45) in the blood [20].

To reproduce the in vivo physiological conditions in vitro, cells are classically maintained in a culture medium containing DMEM, that provides the nutrients and the carbonate ions, and are maintained in a humidified atmosphere of 5% CO${}_{2}$ at 37 °C. This low percentage of CO${}_{2}$ is recommended for cultures with an initially high number of cells or for fast growing cell cultures since cells will produce lactic acid that will rapidly acidify the medium. However if a small number of cells or slow growing cells are considered then an important pH drift towards higher values is often observed and the consequence for the cells are rarely addressed [21,22].

In order to validate our methodology, we measured the pH drift of culture medium using SNARF-4F, BCECF and a pH meter. We considered three initial pH of the culture medium: two acidic pH (5 and 6) and the physiological pH (7.4). For each of these conditions the pH at equilibrium can be calculated from the expression that defined the equilibrium constant ($K_{eq}$) of reaction (3):

(4) $$K_{eq}=\frac{{\left[{HCO}_{3}^{-}\right]}_{eq}\times {\left[H^{+}\right]}_{eq}}{{\left[{CO}_{2}\left(aq\right)\right]}_{eq}}$$

where, [HCO${}_{3}^{-}$]${}_{eq}$ and [CO${}_{2}$(*aq*)]${}_{eq}$ represent the concentrations of HCO${}_{3}^{-}$ and CO${}_{2}$(*aq*) at equilibrium, respectively. We take the value $K_{eq}=8.0\times 10^{-7}$ mol/L, that corresponds to the dissociation of carbonic acid in blood at 37 °C [23], since DMEM is closer to blood plasma than water.

Using the expressions of K${}_{eq}$, it is necessary to know the [HCO${}_{3}^{-}$]${}_{eq}$ and [CO${}_{2}$(*aq*)]${}_{eq}$ to find [H${}^{+}$], or in other words the pH, with Equation (4). However, it is sometimes impossible to experimentally know all the concentrations of the substances present at equilibrium. If one knows the initial concentration of reactants and products, it is possible to predict all equilibrium concentrations algebraically. This is done using an Initial-Change-Equilibrium (ICE) table method. In DMEM, the initial concentration of carbonate ions is [HCO${}_{3}^{-}$]${}_{i}$ = 0.044M and the initial concentration of aquaeous CO${}_{2}$, determined from Henry’s law is [CO${}_{2}$]${}_{i}=K_{C{\mathrm{>O}}_{2}}\times P_{CO_{2}}=1.5\times 10^{-3}$M where the Henry’s constant of CO${}_{2}$ in blood at 37 °C is $K_{CO_{2}}=3\times 10^{-2}$ mole/(L.Atm) [23] and $P_{CO_{2}}=0.05$ Atm (5% CO${}_{2}$).

Table 1 summarizes the predicted pH at equilibrium for each initial pH tested.

**Figure 10:**
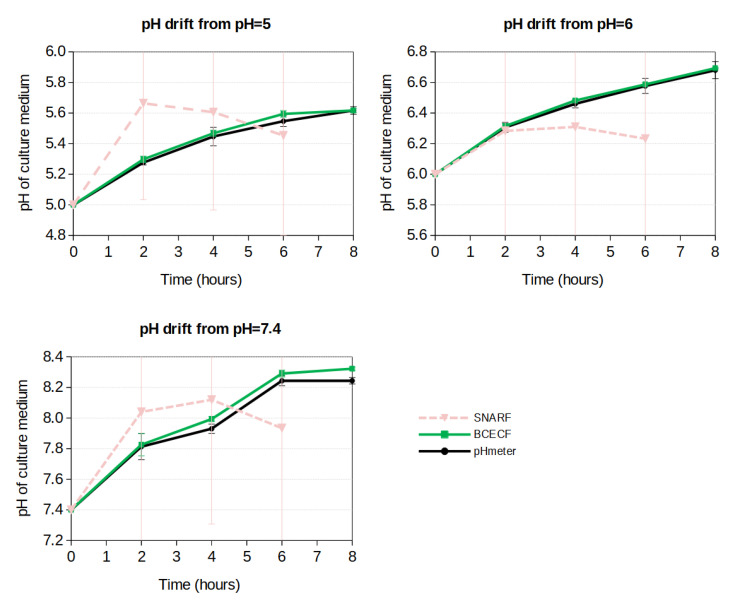
pH drift measured with BCECF and a pH-meter. Measurements with SNARF-4F are outside the range of validity of this probe which explains its poor performance.

**Figure 11:**
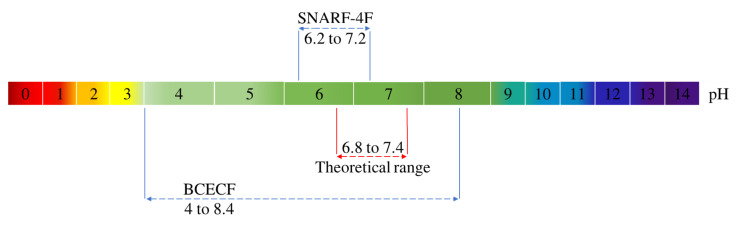
Validity ranges of the BCECF and SNARF-4F probes deduced from their respective calibration curves.

pH drift measurements are presented in Figure 10. The results clearly show that the BCECF probe is very accurate to evaluate the pH with a precision lower than 0.1 pH unit for all the measured pH. On the other hand the SNARF-4F probe exhibits a bad performance since the measurements performed fall outside the validity zone of SNARF-4F (Figure 11). Concerning BCECF our method greatly improves its validity range. The usual theoretical range is for pH within 6.8 and 7.4 but we extended it from 4 to 8.4 according to the BCECF calibration curve (Figure 9).

## 4. Discussion

As part of this study, two fluorescent probes were tested for pH evaluation of cell culture medium. In evaluating these probes, whole emission spectra were analyzed.

SNARF-4F is a fluorescent pH probe that exhibits changes in spectral shape associated with its protonated and deprotonated forms in solution. When excited at 514 nm, solutions predominantly containing the deprotonated form display a deep red long-wavelength band with a maximum situated at 668 nm, which drops and shifts to the yellow-orange upon acidification of the solution. The changes are accompanied by the appearance of a new band peaking at 599 nm, which can be assigned to the fluorescence of the protonated species. This shift between protonated and deprotonated species allows us to use the ratio of fluorescence intensities ($R=I_{599\mathrm{nm}}/I_{668\mathrm{nm}}$) from the probe at two emission wavelengths (599 nm and 668 nm) for more accurate pH determinations. SNARF-4F is therefore used as a single excitation-dual emission probe where the excitation is performed at only one excitation wavelength and fluorescence emission is measured at two emission wavelengths. This approach is called emission ratiometric measurement.

In contrast, BCECF probe does not exhibit significant changes in the spectral shape between the protonated and deprotonated species and the analysis of the probe’s emission spectra shows that the intensity of the emitted light is dependent on the pH as well as the environment of the probe such as the concentration, the photobleaching and the surrounding ions of the probe. Therefore, the use of one excitation wavelength does not make it possible to obtain the fluorescence variations only linked to a change in pH. A second excitation wavelength is then used. This approach is called excitation ratiometric measurement. With two excitations, the manipulation time with the BCECF will be two times longer than that of the SNARF-4F.

Due to material limitations on our confocal microscope, we developed a method to measure the pH with the BCECF probe, based on the available excitation wavelengths. We have found that the two excitation wavelengths 405 nm and 488 nm are two appropriate wavelengths for pH measurements with the fluorescent probe BCECF.

SNARF-4F has been synthesized to be a promising fluorescent sensitive pH probe in the 6–7.5 pH range. This is in agreement with the results that we have obtained after the spectral study of this probe in confocal microscopy. The emission ratiometric measurements with the SNARF-4F probe allowed us to conclude on the sensitivity of this probe in the range of pH 6.2–7.2 (Figure 11).

It was reported that BCECF probe allows measurements in the physiological pH range of 6.8–7.4. In such a case, the typical ratiometric method is to use the two excitation wavelengths 440 nm and 488 nm and fix the emission at 537 nm. Herein lies the distinguishing point of our study. Despite the material limitations on our confocal microscope, the method that we developed using the available excitation wavelengths has allowed us to evaluate the pH drift in culture medium with great precision and to increase drastically the range of validity of the BCECF probe. We found that the use of the two excitation wavelengths 405 nm et 488 nm allows to detect the pH in 4–8.4 pH range (Figure 11).

## Figures and Tables

**Table 1:** pH drift measured with the pH meter after 8 h and predicted pH at equilibrium using the ICE table method. Depending on the initial pH, it takes 9 to 14 h to reach the equilibrium.

Initial pH	pH at 8 h	Predicted pH at Equilibrium
5.00	5.61	6.07
6.00	6.68	6.91
7.40	8.24	8.52

## Data Availability

Not applicable.
